# Curcumin and omega-3 ameliorate experimental osteoarthritis progression in terms of joint pain and mitochondrial dysfunction

**DOI:** 10.1186/s12950-025-00453-x

**Published:** 2025-07-15

**Authors:** JooYeon Jhun, Donghwan Lee, Hyun Sik Na, Keun-Hyung Cho, Seung Yoon Lee, Jeong Su Lee, Young Joon Lee, Seok Jung Kim, Sung-Hwan Park, Mi-La Cho

**Affiliations:** 1 Lab of Translational ImmunoMedicine (LaTIM), Catholic Research Institute of Medical Science, College of Medicine, The Catholic University of Korea, Seoul, 06591 Republic of Korea; 2 Department of Pathology, College of Medicine, The Catholic University of Korea, Seoul, 06591 Republic of Korea; 3 Department of Orthopedic Surgery, Yeouido St. Mary’s Hospital, College of Medicine, The Catholic University of Korea, Seoul, Republic of Korea; 4 Department of Orthopedic Surgery, Uijeongbu St. Mary’s Hospital, College of Medicine, The Catholic University Korea, Seoul, Republic of Korea; 5 Division of Rheumatology, Department of Internal Medicine, Seoul St. Mary’s Hospital, College of Medicine, The Catholic University of Korea, Seoul, Republic of Korea; 6 Department of Medical Sciences, Graduate School of The Catholic University of Korea, Seoul, Republic of Korea

**Keywords:** Osteoarthritis, Monosodium iodoacetate (MIA), Curcumin, Omega-3, Mitochondrial marker

## Abstract

**Background:**

Osteoarthritis (OA), a chronic degenerative disorder, induces pain, joint inflammation, and destruction of the articular cartilage matrix. Curcumin and omega-3 have been used as dietary supplements for OA due to their anti-inflammatory and antioxidant properties. However, there is no evidence demonstrating a synergistic effect in OA. The current study aimed to investigate the therapeutic effects and underlying mechanism of a combination of curcumin and omega-3 in the treatment of OA.

**Methods:**

Wistar rats were injected with monosodium iodoacetate to induce OA. Oral treatments of a vehicle, curcumin, curcumin and omega 3, or celecoxib were administered. Pain was analyzed according to the paw withdrawal latency, paw withdrawal threshold, and weight bearing ability. The joint was isolated from OA rats, and cartilage damage was evaluated using histomorphological techniques, the Mankin scoring system, and micro computed tomography analysis. Protein expression in the joint was examined using immunohistochemistry. The expression levels of catabolic markers were measured in curcumin and omega-3-treated OA chondrocytes.

**Results:**

The OA animal model revealed diminished pain and cartilage conservation in response to the combined treatment. mRNA levels of matrix metalloproteinase 1 (MMP1), MMP3, and MMP13 were reduced in interleukin-1 beta-simulated human OA chondrocytes. Additionally, mitochondrial markers, cytochrome c oxidase 4, and TOMM20, were increased by the combination treatment.

**Conclusions:**

These findings suggest promising therapeutic outcomes for the combined treatment of curcumin and omega-3 in OA patients.

## Background

Increasing attention is being paid to degenerative diseases due to medical developments and an increased lifespan in modern society [1]. As such, osteoarthritis (OA), one of the most common degenerative diseases caused by aging, is seeing a rise in incidence, as evidenced by the numerous studies seeking treatment for this condition [2, 3]. OA is a disease that progresses through cartilage destruction and low-grade joint inflammation [4]. Mitochondrial dysfunction has been identified as a major cause of OA in relation to aging. The mitochondrial respiratory chain (MRC) has low activity, and the mitochondrial membrane potential is reduced in the chondrocytes of OA patients. As a result, there is an increase in the generation of inflammatory mediators and reactive oxygen species (ROS), as well as materials that induce matrix destruction. This mitochondrial dysfunction causes chondrocyte aging and facilitates terminal differentiation. As a result, chondrocyte apoptosis, matrix catabolism, and cartilage matrix calcification occur, leading to OA progression [5–7]. 

Although aging occurs in all organs, OA is one of the most common degenerative diseases, indicating that chondrocytes are particularly vulnerable to such a change. Previous studies have shown that aging-induced mitochondrial dysfunction leads to chondrocyte destruction; that said, few studies have identified a treatment [8, 9]. 

Curcumin is a substance extracted from *Curcuma species* and is commonly referred to as turmeric. It has long been used as a medicinal ingredient and a spice in Asia and it is known to have proven stability [10, 11]. As curcumin exhibits antioxidant and anti-inflammatory properties, it has been studied as a therapeutic agent for various inflammatory diseases. Previous in-vitro studies have shown that curcumin inhibits the NF-κB pathway, the release of proteoglycans and metalloprotease, and the expression of inflammatory mediators, thus reducing chondrocyte apoptosis. Clinical trials have been conducted on curcumin treatment that have demonstrated its efficacy to some extent [12]. In addition, recently it has been proven that curcumin helps to restore mitochondrial membrane potential and reduce mitochondrial damage. Consequently, curcumin is noted for its value as a therapeutic agent through recovery of mitochondrial dysfunction [11]. 

Omega-3 is well known, particularly as a component of fish oil, and its efficacy has been demonstrated in studies of insulin sensitivity, glucose homeostasis, and cardiovascular disease [13, 14]. From a perspective similar to that described for curcumin, omega-3 has been studied as a therapeutic agent for OA treatment. A number of animal studies have shown that omega-3 reduces the expression of inflammatory markers, protects cartilage, and reduces pain [15, 16]. In clinical studies applying omega-3 therapy to OA patients, there is still a lack of studies showing clear improvement [17]. Omega-3 treatment has also been noted for its ability to improve mitochondrial function in numerous studies. The administration of omega-3 changes the mitochondrial membrane lipid composition, affecting mitochondrial bioenergetics. Thus, improved mitochondrial function can be expected [13, 18]. 

Given the efficacy of both curcumin and omega-3 as therapeutic agents, we hypothesized that an improved treatment effect could be gained from the combined administration of the two. Monosodium iodoacetate (MIA)-induced OA rat models were used in the experiment. The rats were assigned to one of four groups: the vehicle (control) group; the celecoxib group (receiving a non-steroidal anti-inflammatory drug); the curcumin group (receiving a single administration of curcumin); or the curcumin + omega-3 group (received a combined administration of curcumin and omega-3). The anti-nociceptive effect was analyzed. The Osteoarthritis Research Society International (OARSI) score and Mankin score were evaluated in histologic analyses, and bone protection in micro-computed tomography (micro-CT) bone images were compared and analyzed. OA patient-derived chondrocytes were treated with interleukin-1 beta (IL-1β) to create an OA environment, and then the expression of catabolic markers and inflammatory mediators was confirmed. We conducted the curcumin therapy and curcumin + omega-3 therapy on the MIA-induced OA rat model and measured cytochrome c oxidase 4 (COX IV) and Tomm20, both mitochondrial markers, to prove the effects of the treatment in improving mitochondrial dysfunction.

## Methods

### Animals

Seven-week-old male rats were purchased from Central Laboratory Animal, Inc. (Seoul, Korea). The rats were housed three per cage in a room with temperature control (21℃–22℃) and a 12 h/12 h light/dark cycle. All animals had access to sterile food and water. The rats were randomized into four groups of six rats each. All of the study procedures were approved by the Animal Research Ethics Committee of The Catholic University of Korea (2018-0174-04).

### MIA-induced OA and treatment

Rats were randomly assigned to treatment groups (curcumin, curcumin + omega-3, or celecoxib group) or the control (vehicle) group before the study began. After anesthetization with isoflurane, the rats (*n* = 6 per group) were injected with 3 mg MIA (Sigma-Aldrich, St. Louis, MO, USA) in the intra-articular space of the right knee through the patellar ligament, using a 26.5-G needle. Celecoxib was provided by Hanlim Pharm (Seoul, Korea). Curcumin (100 mg/kg), omega-3 (110 mg/kg), and celecoxib (30 mg/kg) were administered orally every day starting from day 3 after MIA induction and continued for 21 days. The control rats were given an equivalent of corn oil. The rats were sacrificed at day 21 from MIA injection.

### Osteoarthritis pain assessment

Mechanical sensitivity was used to assess pain, as described previously [19, 20]. Following MIA injection, a dynamic plantar aesthesiometer (Ugo Basile, Gemonio, VA, Italy) was used to evaluate the response. Von Frey hair was used for mechanical sensitivity assessment. Pain was scored based on previous stimulation of the masseter, using rigid von Frey filaments and a force transducer (model 2290; Electronic von Frey, IITC, Inc., Woodland Hills, CA, USA). The force required to elicit hind-paw withdrawal was recorded three times following stimulations at 1-min intervals. The meaning of the three values was used for analysis.

### Weight balance assessment

Weight bearing was evaluated using an incapacitance tester (Linton Instrumentation, Norfolk, UK) that included a dual-channel weight mean value. The rats were positioned in a plastic chamber. The strength applied by an individual hind limb was averaged over more than a 3-s time period. Individual data points were the average of three measurements. The percentage of weight on the handled (ipsilateral) hind limb was calculated utilizing the following equation: (weight on right leg/weight on right and left legs) × 100.

### Histopathological analysis

Histological changes were analyzed to determine the effects of curcumin, and the combination treatment of curcumin + omega-3 administration in the knee joints of rats. The rats were perfused via the ascending aorta with 10% neutral buffered formalin. The knee joints, including the patella and joint capsule, were resected and maintained in the same fixative for an additional 48 h at 4 °C. The fixed specimens were decalcified with 5% formic acid decalcifier for 6 days at 4 °C. After decalcification, the specimens were embedded in paraffin. Standardized 7-mm serial sections were obtained at the medial and lateral midcondylar level in the sagittal plane and stained with hematoxylin and eosin (H&E). A modified Mankin’s score was used to classify histological injury of the articular cartilage, as follows: = normal; 1 = irregular surface, including fissures into the radial layer; 2 = pannus; 3 = absence of superficial cartilage layers; 4 = slight disorganization (cellular row absent, some small superficial clusters); 5 = fissure into the calcified cartilage layer; and 6 = disorganization (chaotic structure, clusters, and osteoclast activity). Cellular abnormalities were scored on a scale ranging from 0 to 3, where 0 = normal; 1 = hypercellularity, including small superficial clusters; 2 = clusters; and 3 = hypocellularity. Matrix staining was scored on a scale ranging from 0 to 4, where 0 = normal/slight reduction in staining; 1 = staining reduced in the radial layer; 2 = staining reduced in the interterritorial matrix; 3 = staining present only in the pericellular matrix; and 4 = staining absent. The joint space width was estimated based on the sum of the nearest distance of the medial and lateral tibiofemoral joints. Histological evaluations were performed independently by two experienced researchers who were blinded to the study groups.

### Immunohistochemistry

Immunohistochemistry slides were deparaffinized and rehydrated using a graded ethanol series. The sections were incubated overnight at 4 °C with antibodies to COX IV (Santa Cruz Biotechnology, Santa Cruz, CA, USA) and Tomm20 (Abcam, Cambridge, UK). The slides were then treated with secondary antibodies and biotinylated anti-mouse IgG for 20 min, conjugated to a streptavidin peroxidase complex (Vector Laboratories, Burlingame, CA, USA) for 1 h, and then treated with 3,30-dia-minobenzidine (Dako, Glostrup, Denmark). The slides were counterstained with Mayer’s hematoxylin and photographed using a photomicroscope (Olympus, Tokyo, Japan).

### In-vivo micro-computed tomography imaging and analysis

Micro-CT imaging and analysis were performed using a bench-top cone-beam-type in-vivo animal scanner (mCT 35; SCANCO Medical, Bruttisellen, Switzerland). The animals were imaged at settings of 70 kVp and 141 µA using an aluminum 0.5-mm-thick filter. The pixel size was 8.0 μm, and the rotation step was 0.4 °C. Cross-sectional images were reconstructed using a filtered back-projection algorithm (NRecon software, Bruker Micro CT, Kontich, Belgium). For each scan, a stack of 286 cross-sections were reconstructed at 2000 × 1335 pixels. The bone volume and surface were analyzed at the femur.

### Primary culture and treatment of OA chondrocytes

All relevant protocols were approved by the Institutional Review Board of Uijeongbu St. Mary’s Hospital (HC14TISI0071) and performed in accordance with the Declaration of Helsinki. All patients provided written informed consent. OA was diagnosed using the American College of Rheumatology criteria [21]. Isolation of human chondrocytes was performed as described previously [12]. Briefly, chondrocytes were isolated from the cartilage of patients. Cartilage was digested with 0.5 mg/mL hyaluronidase, 5 mg/mL protease type XIV, and 2 mg/mL collagenase type V. Finally, chondrocytes were incubated in Dulbecco’s modified Eagle medium (DMEM), including 10% fetal bovine serum. The isolated human OA chondrocytes of passage 3 were cultured in the presence or absence of IL-1β (20 ng/mL), curcumin (100 μm), DHA (Docosahexaenoic acid), (50 μm), and celecoxib (10 μm).

### Enzyme-linked immunosorbent assay

The concentrations of MCP-1 in culture supernatants were measured using a DuoSet enzyme-linked immunosorbent assay (ELISA) kit (R&D Systems, Minneapolis, MN, USA). The 96-well plates (Nunc, Roskilde, Denmark) were coated with capture antibodies for anti-human monocyte chemoattractant protein-1 (MCP-1, R&D Systems) and incubated overnight at 4 °C. After the overnight incubation, the plates were blocked with phosphate-buffered saline containing 1% bovine serum albumin and 0.05% Tween 20 for 2 h at room temperature. Cell culture supernatants were added to the plates and incubated at room temperature for 2 h. Subsequently, the plates were washed, detection antibodies were then added, and the reaction mixtures were incubated for 2 h at room temperature. The plates were washed again and then incubated with streptavidin-horseradish peroxidase for 20 min. Following an additional wash step, the substrate solution was added for incubation for 20 min. The stop solution was then added. The results were analyzed by determining the absorption at 405 nm (A405).

### Real-time polymerase chain reaction

Total RNA was extracted using TRI Reagent (Molecular Research Center, Cincinnati, OH, USA) according to the manufacturer’s instructions. Complementary DNA (cDNA) was prepared by reverse transcription of single-stranded RNA using a high-capacity cDNA reverse transcription kit (Applied Biosystems, Foster City, CA, USA), according to the manufacturer’s instructions. Polymerase chain reaction (PCR) amplification was performed using a LightCycler 2.0 instrument (software version 4.0; Roche Diagnostics, Indianapolis, IN, USA). All reactions were conducted using LightCycler FastStart DNA Master SYBR Green I (TaKaRa, Shiga, Japan), according to the manufacturer’s instructions. The primer pairs used were as follows: control human gene β-actin, 5′-GGA CTT CGA GCA AGA GAT GG-3′ (sense) and 5′-TGT GTT GGC GTA CAG GTC TTT G-3′ (antisense); human MMP-1, 5′-CTG AAG GTG ATG AAG CAG CC-3′ (sense) and 5′-AGT CCA AGA GAA TGG CCG AG-3′ (antisense); MMP-3, 5′-CTC ACA GAC CTG ACT CGG TT-3′ (sense) and 5′-CAC GCC TGA AGG AAG AGA TG-3′ (antisense); MMP-13, 5′-CTA TGG TCC AGG AGA TGA AG-3′ (sense) and 5′-AGA GTC TTG CCT GTA TCC TC-3′ (antisense). All expression values were normalized to that of β-actin mRNA. PCR amplification and analysis were performed using a LightCycler real-time PCR system (Roche Holding AG, Basel, Switzerland).

### Statistical analysis

Statistical analyses were performed using the nonparametric Mann–Whitney U-test for comparisons between two groups, and one-way analysis of variance with the Bonferroni post-hoc test for multiple comparisons. GraphPad Prism (ver. 9.2.0; GraphPad Software Inc., San Diego, CA, USA) was used for all analyses. The data are presented as the mean ± standard deviation. For all comparisons, *P* < 0.05 was taken to indicate statistical significance.

## Results

### Curcumin and omega-3 suppress the pain threshold in MIA-induced OA rats

**Fig. 1:**
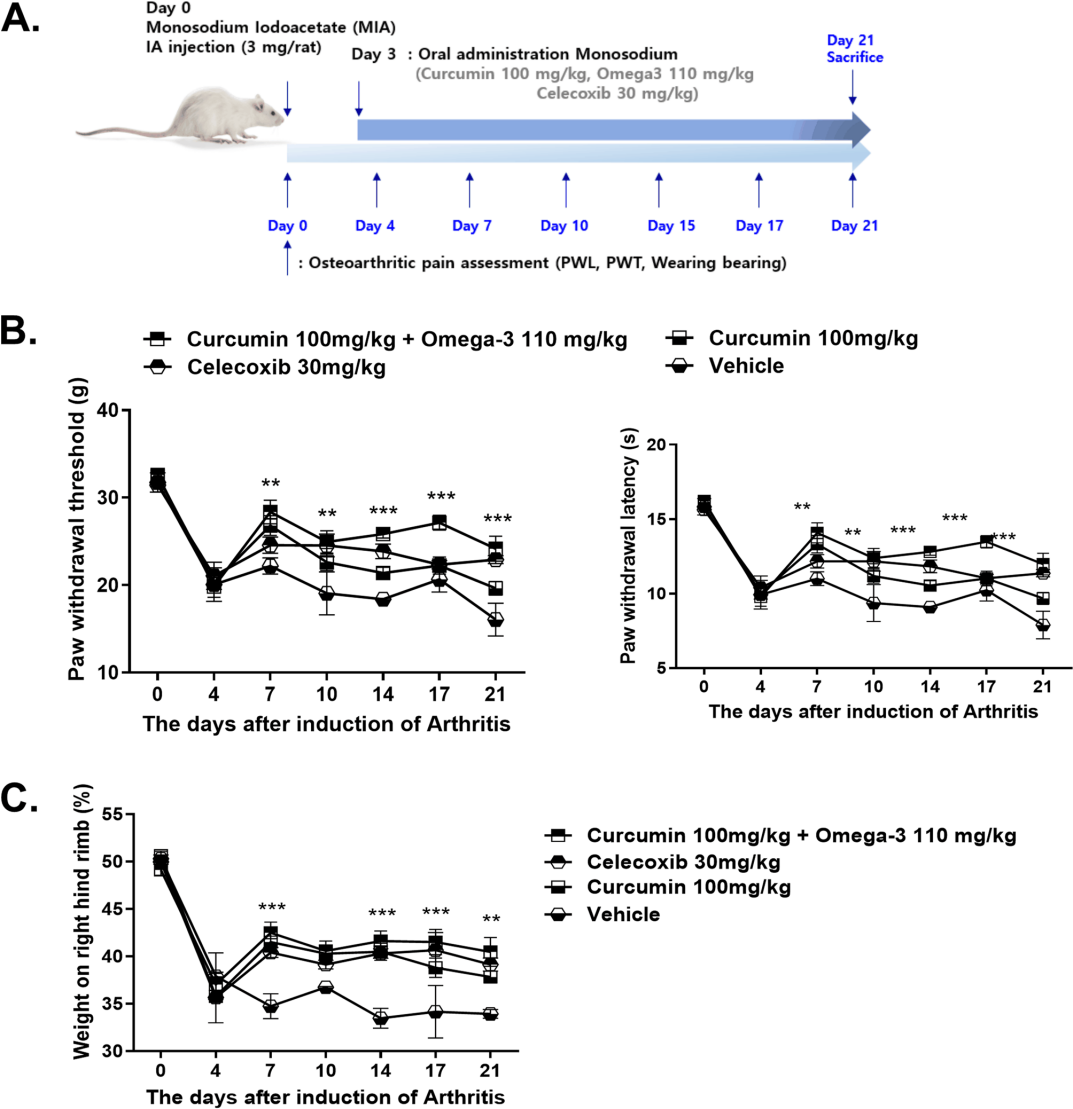
Therapeutic effects of curcumin and omega-3 treatment in a model of monosodium iodoacetate (MIA)-induced osteoarthritis (OA) in rats. **A** Schedule of rat experimental procedure is shown by a diagram. **B** Rats were injected with 3 mg of MIA, in the right knee. The drug administration was orally administered daily for 21 days after MIA injection. Behavioral tests of mechanical hyperalgesia were performed using a dynamic plantar aesthesiometer. Pain behavior was analyzed as paw withdrawal latency (left) and paw withdrawal threshold (PWT) (right) in vehicle-treated (*n* = 6), curcumin-treated (*n* = 6), a combination of curcumin and omega-3-treated (*n* = 6), and celecoxib-treated (*n* = 6) MIA-induced rats. **C** Weight bearing was measured in all groups. Data are presented as the mean ± standard deviation (SD). **p < 0.01; *** *p* < 0.001

We administered curcumin alone and both curcumin and omega-3 to MIA-induced OA rats to prove that curcumin and omega-3 can help regulate pain. Paw withdrawal threshold (PWT), paw withdrawal latency (PWL), and weight bearing were measured and the two groups were compared to that in which celecoxib, an NSAID, was administered. Using a dynamic plantar aesthesiometer, PWT and PWL were measured for the four groups of vehicle, celecoxib, curcumin, and a combination of curcumin and omega-3 (curcumin + omega-3). After OA was induced by MIA only on the right side, the weight bearing of both paws was measured (Fig. 1A). The PWT and PWL measurements of the curcumin group, the celecoxib group, and the curcumin + omega-3 group were compared with those of the vehicle group, and an anti-nociceptive effect was confirmed. The curcumin + omega-3 group showed a larger increase in PWT and PWL than the celecoxib group; this indicates that the combination of curcumin and omega-3 provided more effective pain regulation than celecoxib (Fig. 1B). The curcumin group, the celecoxib group, and the curcumin + omega-3 group showed greater weight bearing ability than the vehicle group; thus, the pain decreased for all three groups (Fig. 1C).

### Curcumin and omega-3 reduce cartilage destruction in MIA-induced OA rats

**Fig. 2:**
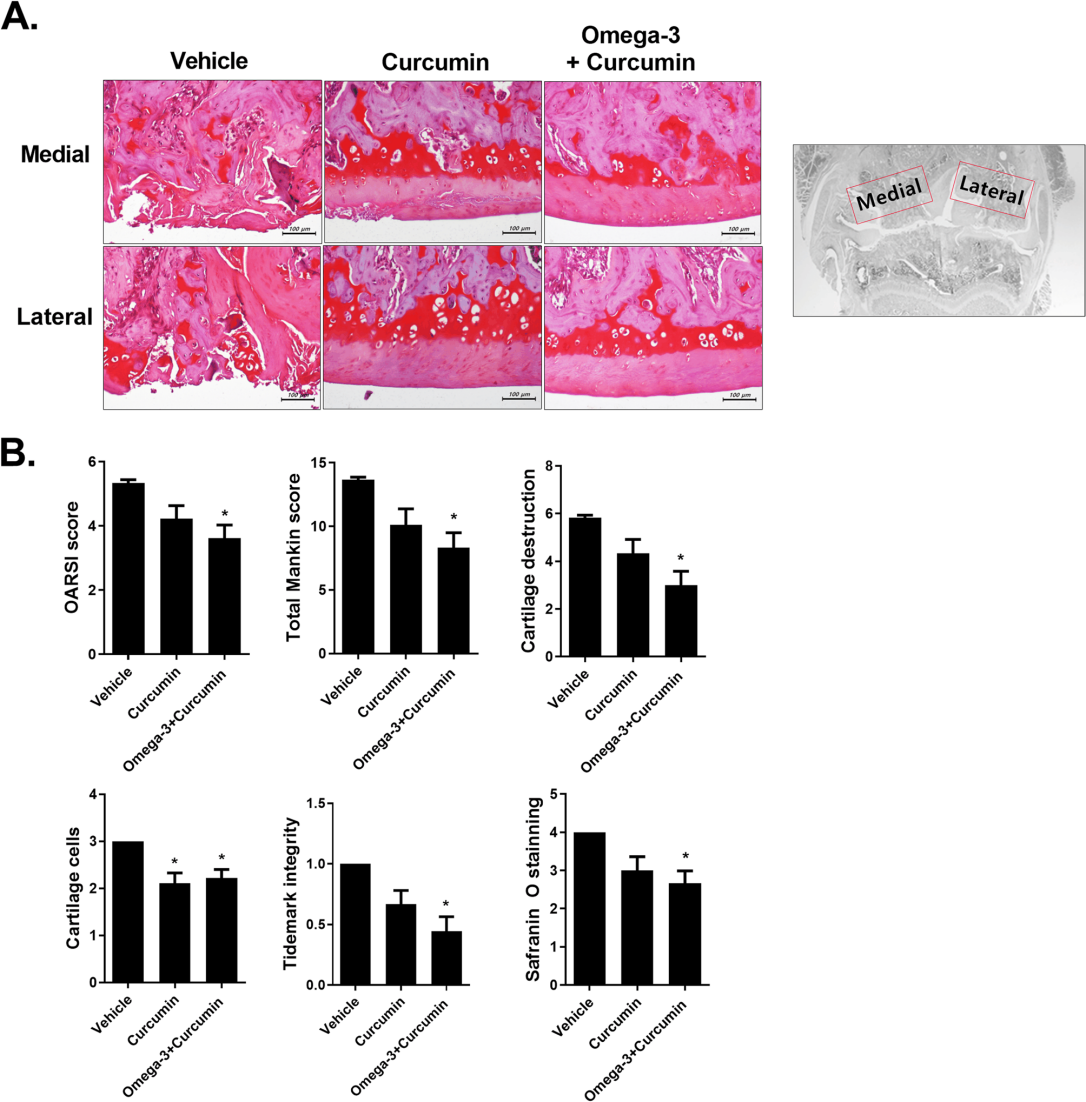
Effects of curcumin and omega-3 in the MIA-induced OA model. **A** The knee joints of OA rats treated with either vehicle, curcumin, a combination of curcumin and omega 3, or celecoxib were stained with hematoxylin and eosin. **B** Data are presented as the mean ± SD. *p < 0.05

Knee joint tissues were collected from three groups—the vehicle group, the curcumin group, and the curcumin + omega-3 group, and safranin O staining was conducted (Fig. 2A). To compare the cartilage protection effect, each group was evaluated using their OARSI scores and total Mankin scores. Both the OARSI score and the total Mankin score were lower in the curcumin group and the curcumin + omega-3 group than in the vehicle group, and both scores were lower in the curcumin + omega-3 group than in the curcumin group. Among the parameters of the Mankin score, the curcumin + omega-3 group showed lower scores ​​than the curcumin alone in cartilage destruction, tidemark integrity, and safranin O staining. For measurement of cartilage cells, the scores were similar among groups (Fig. 2B). The results showed that both the curcumin therapy and the curcumin + omega-3 therapy were effective in inhibiting cartilage destruction and that the combined administration of curcumin and omega-3 was more effective than the single administration of curcumin.

### Curcumin and omega-3 reduce bone protection in MIA-induced OA rats

**Fig. 3:**
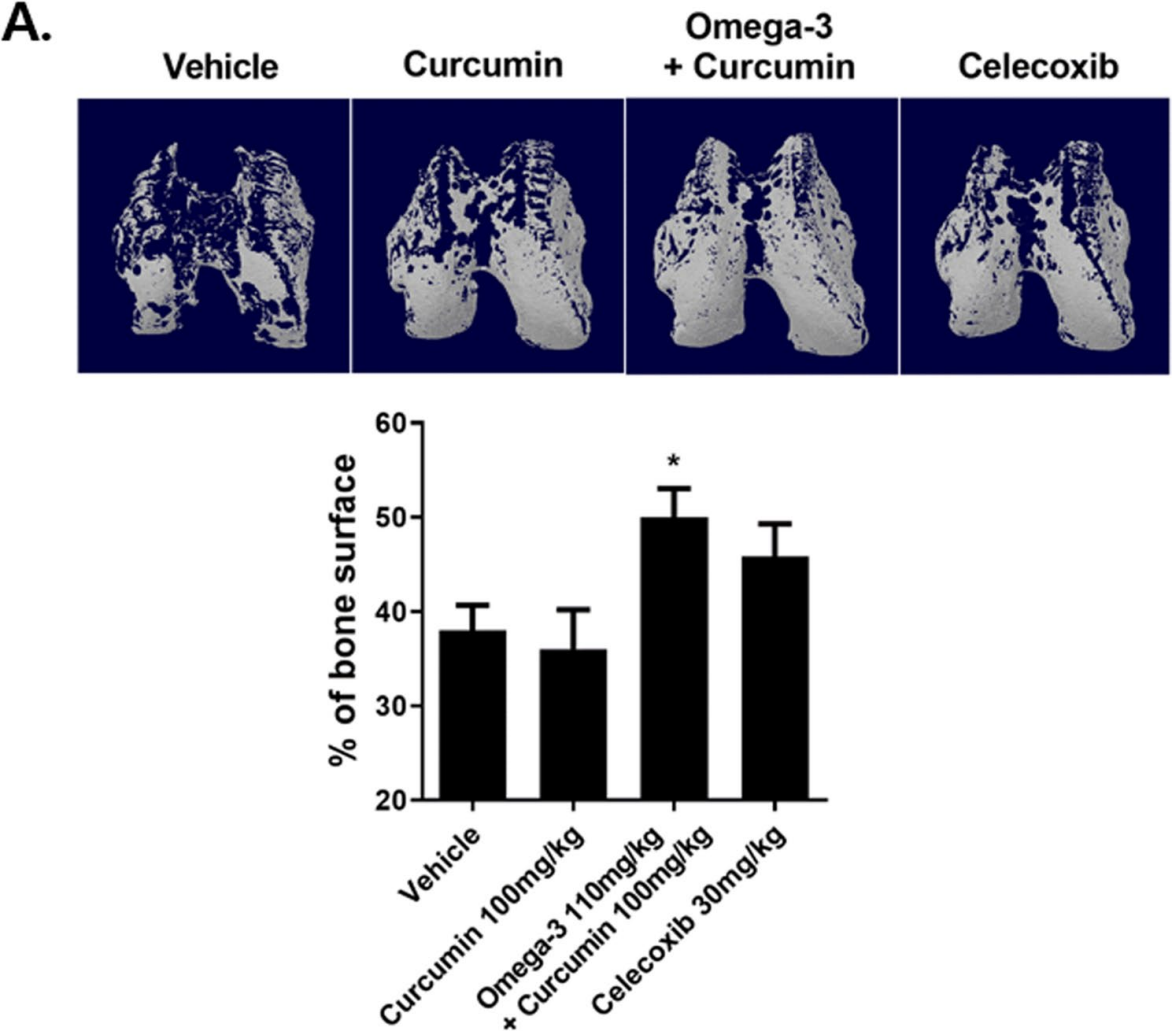
Curcumin and Omega-3 treatment decrease bone protection in MIA rats. **A** Bone samples were collected from the MIA-induced right knee joint at 21 days. Joints were scanned using micro computed tomography. The bone surface was measured to Bone J. The data show the mean ± SD of three independent experiments. *p < 0.05

Micro-CT was used to confirm that the combined administration of curcumin and omega-3 reduces bone protection for MIA-induced OA rats. We analyzed micro-CT bone images from the four groups of vehicle, celecoxib, curcumin, and curcumin + omega-3 (Fig. 3A). The bone surface of the curcumin group was similar to that of the vehicle group, yet it was higher in the celecoxib and the curcumin + omega-3 groups, with the curcumin + omega-3 group showing the highest result (Fig. 3B). This implies that the combined administration of curcumin and omega-3 reduces bone protection, even more effectively than celecoxib.

### Curcumin and omega-3 reduce the levels of catabolic markers and inflammatory mediators in human chondrocytes

**Fig. 4:**
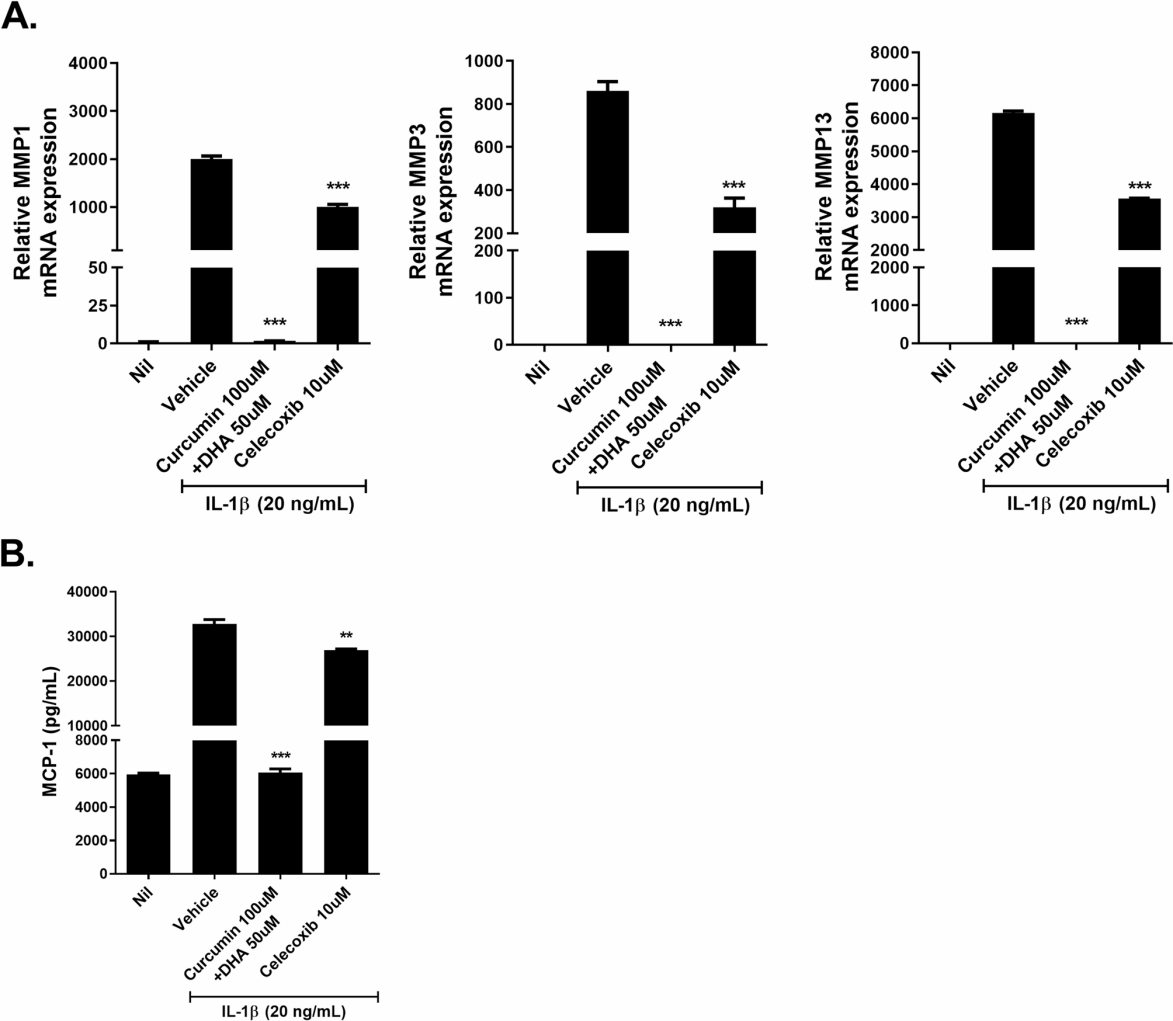
Curcumin and omega-3 treatment attenuates catabolic factor and inflammatory cytokine in human OA chondrocytes. **A** mRNA levels of MMP-1, MMP3, and MMP-13 in human OA chondrocytes were measured using real-time polymerase chain reaction analysis. **B **The concentration of MCP-1 in culture supernatants was measured by an enzyme-linked immunosorbent assay. Data are presented as the mean ± SD of three independent experiments. **p < 0.01; *** *p* < 0.001

Human chondrocytes were treated with IL-1β to induce an OA environment and then the curcumin + omega-3 therapy was performed to compare the effects with those of the celecoxib therapy. The mRNA expression of MMP-1, −3, and − 13, all of which are catabolic markers, were measured in four groups: the vehicle, a mixture of curcumin and DHA, and celecoxib using quantitative PCR. The mRNA expression level of MMP-1, −3, and − 13 in the celecoxib group showed a slight decrease in relation to the vehicle group, whereas a much greater decrease was observed for the curcumin + DHA group. These results imply that the curcumin + DHA therapy can inhibit the catabolic response even more effectively than the celecoxib therapy (Fig. 4A). The expression of monocyte chemoattractant protein-1 (MCP-1), which acts as an inflammatory mediator, was measured with ELISA. Similar to the results above, there was a much larger decrease in the expression of MCP-1 in the curcumin + DHA group than in the vehicle and celecoxib group (Fig. 4B). These results imply that curcumin + DHA therapy effectively inhibits inflammation in human chondrocytes.

### Curcumin and omega-3 restore mitochondrial function in the knee joint of MIA-induced OA rats

**Fig. 5:**
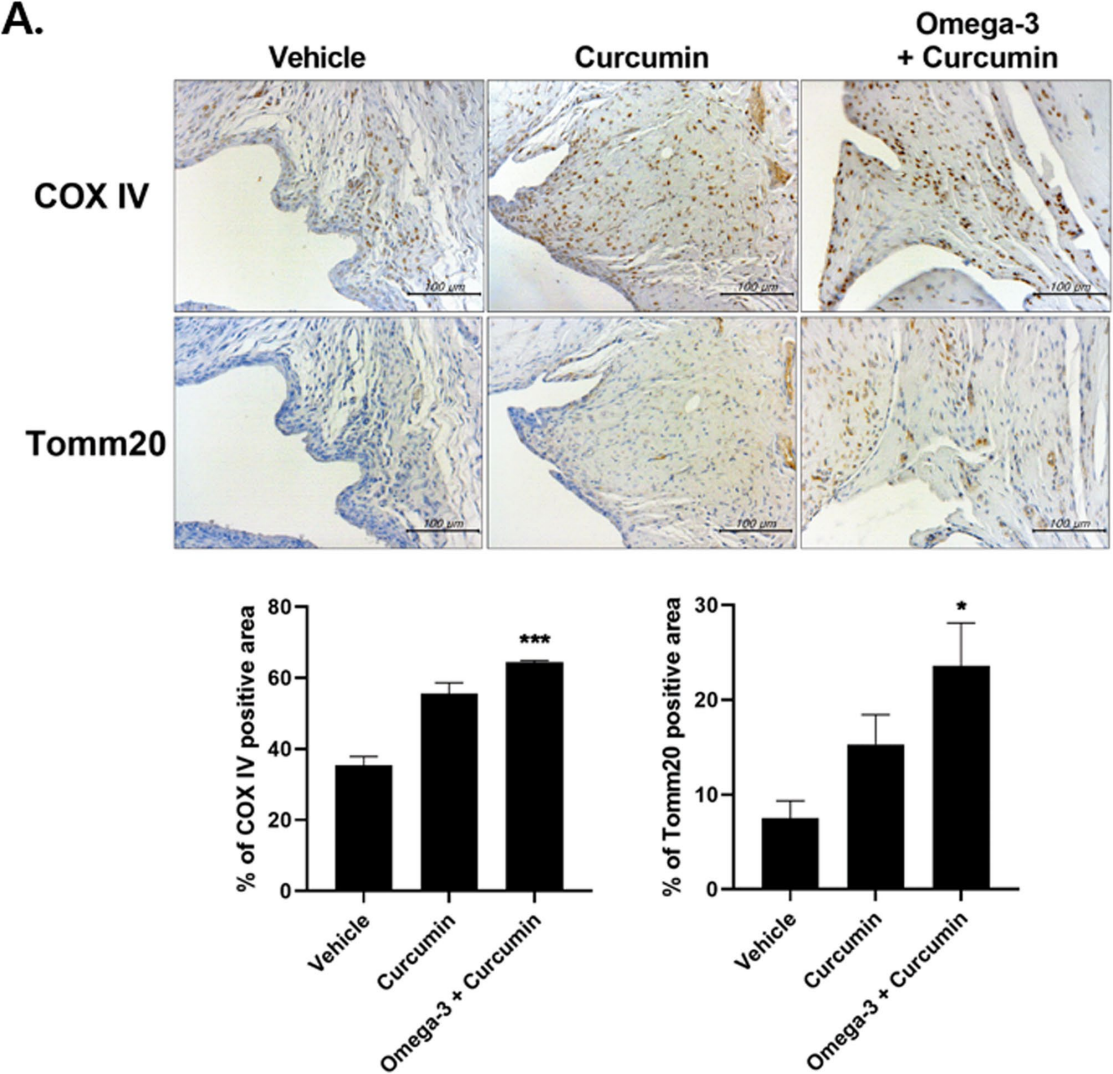
Curcumin and omega-3 treatment increase the mitochondrial function factor in OA synovium. Immunohistochemically (IHC) staining was used to detect the expression levels of cytochrome c oxidase 4 (COXIV) and Tomm20 in the synovium of OA rats. The IHC results revealed that curcumin and omega-3 administration restored MIA-induced OA loss compared to the control. Data are presented as the mean ± SD. *p < 0.05; *** *p* < 0.001

Knee joint tissues were collected from three groups—the vehicle group, the curcumin group, and the curcumin + omega-3 group of MIA-induced OA rats—and immunohistochemistry staining was performed. COX IV and Tomm20 positive areas, both mitochondrial markers, were measured to evaluate the recovery of mitochondrial function (Fig. 5A). The positive area of both mitochondrial markers were larger in the curcumin group and curcumin + omega-3 groups than in the vehicle group. Also, the positive area of both mitochondrial markers were measured to be larger in the curcumin + omega-3 group than in the curcumin group. These results indicate that curcumin helps in the recovery of mitochondrial function in OA and that the combined administration of curcumin and omega-3 is more effective than the single administration of curcumin in the recovery of mitochondrial function (Fig. 5B).

**Fig. 6:**
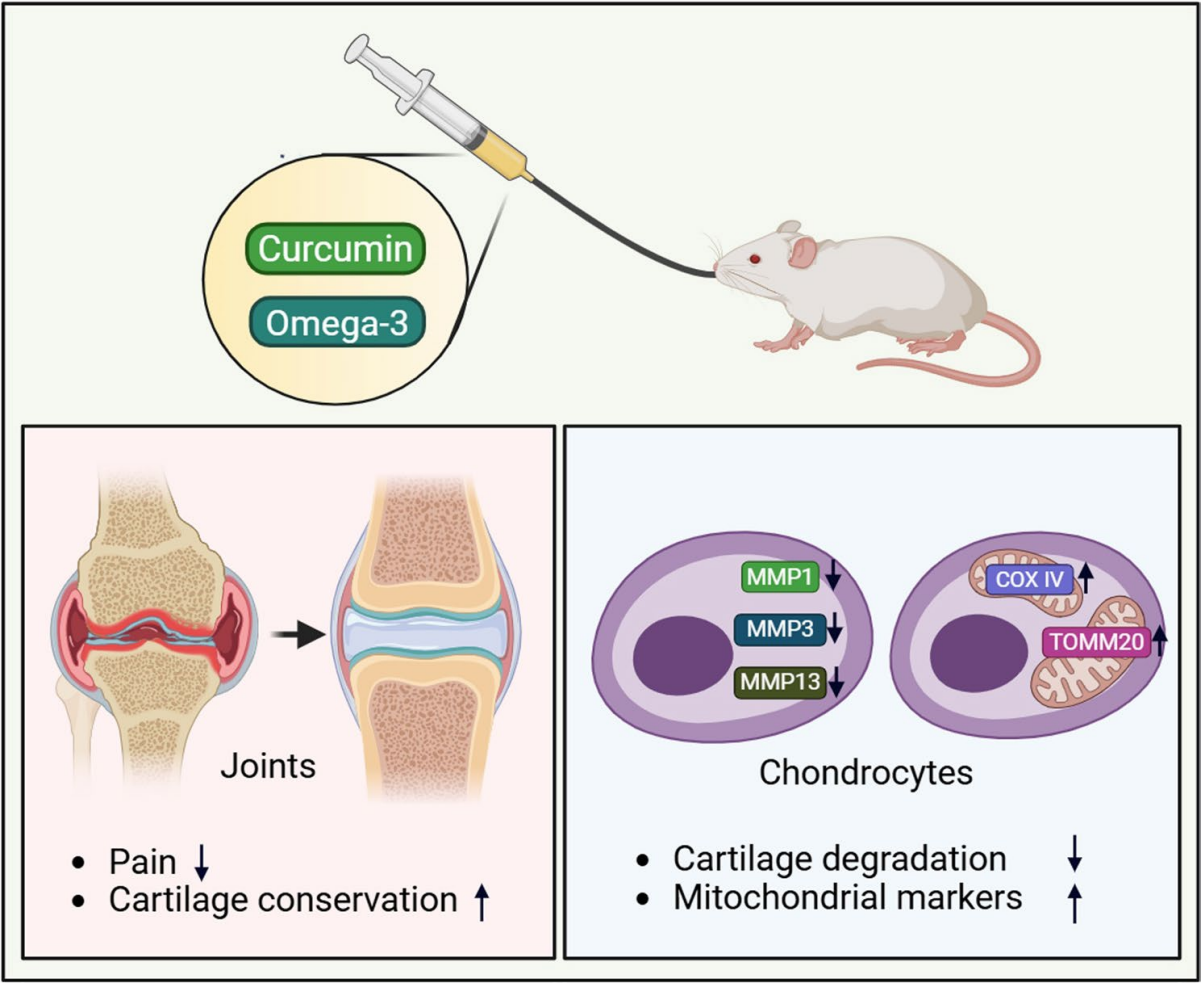
Schematic diagram of therapeutic effect of curcumin and omega-3 on OA through regulation of cartilage degradation and mitochondrial markers. Combined administration of curcumin and omega-3 leads to decreased pain and cartilage destruction in joints of MIA-induced OA rats. In chondrocytes, combined administration leads to a decrease in catabolic factors (MMP-1, MMP-3, MMP-13) and an increase in mitochondrial markers (COX IV, TOMM20). Combined administration of curcumin and omega-3 suppresses both the expression of OA-catabolic factors and increases mitochondrial markers The English in this document has been checked by at least two professional editors, both native speakers of English. For a certificate, please see: http://www.textcheck.com/certificate/Y4J4SU The above statement is here to inform reviewers—who may not be native speakers of English—that the English in this document has been professionally checked. If the link to the certificate above is deleted and copied into a letter then the reviewers will not see it. We STRONGLY recommend that no changes are made. Textcheck should be the last step before final formatting. Typically, authors’ changes result in errors in the English, not improvements. If any text has been misunderstood then please REWRITE the sentence(s) that you need to change, using this file, SAVE, and upload a clean (unmarked) final version of the document to: http://www.textcheck.com/client/submit for a final check. Please do not mark your changes (no highlighting, colored font, bold, etc.) and do not include notes, questions, or comments. Changes that are not because of misunderstanding may incur additional charges; please see: http://www.textcheck.com/text/page/revisions. Requests for a final check should be made within 1 month. For more detailed information, please see: ‘When you receive your completed document’ at http://www.textcheck.com/text/page/guidelines. REVISED DOCUMENTS: A previously checked document that needs changed and new sentences checked (such as post review) is termed a ‘Revised Document’ (http://www.textcheck.com/text/page/fees*).* Revised Documents should be uploaded via ‘Submit Document’ in your online account, with a note that the file is a revision of ‘23,021,614’. Please do not mark your changes; we will use MS Word to compare the document with the most recent complete previous version in your account. When doing so, we cannot consider extracts or versions earlier than the most recent previous version. It is therefore important to upload complete documents. The fee for a Revised Document is based on the wordcount of ALL new and changed sentences. We do not accept new or revised documents on the basis of requests to ‘check only marked text’

## Discussion

As a major source of ROS, mitochondria have long been considered to play an important role in aging. Recent studies have found that the role of ROS in aging is not clear; however, mitochondrial dysfunction has been shown to be strongly linked to aging [22]. 

OA is a degenerative disease associated with aging that causes mitochondrial dysfunction. When the activity of MRC complexes I, II, III, and V is reduced, accordingly the secretion of inflammatory cytokines from chondrocytes and synoviocytes increases, and ROS production becomes elevated. This causes an inflammatory pathological condition of the joints [23, 24] and increases oxidative stress. The inhibition of MRC complex V disturbs autophagy and causes chondrocyte apoptosis [25]. As mitochondrial function decreases, the amount of matrix degradative enzymes increase and the synthesis of extracellular matrix proteins such as collagen and proteoglycan decrease. As a result, cartilage degradation progresses, and OA pathologies such as cartilage matrix calcification and matrix breakdown occur [26, 27]. As seen above, mitochondrial dysfunction acts as a major risk factor of OA. Consequently, a therapeutic approach targeting the recovery of mitochondrial function can be a new OA treatment method [7, 28]. 

As mentioned above, curcumin is a substance extracted from *Curcuma species* and is studied as a therapeutic agent for various inflammatory diseases, due to its antioxidant and anti-inflammatory effects. Curcumin also acts as a direct scavenger of ROS and exhibits a cytoprotective effect through nuclear factor-erythroid-2-related factor 2 (Nrf2). This process has been found to be closely related to mitochondria [29]. Through that process, curcumin enhances mitochondrial membrane potential and increases mitochondrial respiration. In this way, curcumin protects mitochondrial dysfunction and is noted for its efficacy in preventing progression to pathological conditions [11]. 

Many animal studies and human clinical studies have been conducted on curcumin, proving its stability. In some studies, curcumin was applied in combination therapy with other therapeutic agents for OA treatment. For example, curcumin has been administered in combination with glucosamine and with NSAIDs in some studies, with no adverse effect. Curcumin is known to have less adverse effects than NSAIDs. There have been attempts to develop combination therapies for increasing the clinical efficacy of curcumin; this is an area that we continue to explore, with a focus on improving mitochondrial function [12, 30–32]. Omega-3 changes the mitochondrial membrane lipid composition and affects mitochondrial bioenergetics, thus improving mitochondrial function [13, 18]. In this regard, we expect the combination therapy of curcumin and omega-3 to be effective.

In the current study, to confirm the anti-nociceptive effect of the curcumin + omega-3 combination therapy, an experiment was conducted on rat models in which MIA was injected into knee joints to induce OA. To compare the effects with curcumin alone and NSAID treatment, the PWT, PWL, and weight bearing were measured for the four groups of the vehicle, celecoxib, curcumin, and curcumin + omega-3 treatments. The curcumin + omega-3 group was more effective for pain regulation than the curcumin or celecoxib groups alone. Next, to evaluate cartilage destruction, curcumin therapy and curcumin + omega-3 therapy were performed on the MIA-induced OA rat model and compared with the vehicle, in which the OARSI score and the total Mankin score were measured. Both scores were lower in the curcumin + omega-3 group than in the curcumin alone group; this indicates that the combined administration was more effective than the single curcumin administration in inhibiting OA progression. We analyzed micro-CT images of the bone surface. The bone volume was higher in the curcumin + omega-3 group than with curcumin or celecoxib alone. Thus, the combined administration was more effective in inhibiting bone protection in the OA rat model.

Human chondrocytes were treated with IL-1β to induce an OA environment and were then treated with curcumin and omega-3. The mRNA expression of MMP-1, −3, and − 13, all catabolic markers, and the expression of MCP-1, which is an inflammatory mediator, were measured in the four groups of NIL, vehicle, a mixture of curcumin and DHA, and celecoxib. All four markers decreased sharply in the curcumin + DHA group compared to the vehicle and celecoxib groups. These results imply that DHA can effectively inhibit the catabolic response and inflammation in chondrocytes in an OA environment. Next, to evaluate the recovery of mitochondrial function, an experiment was conducted using the MIA-induced OA rat model. After conducting the experiment with the vehicle, curcumin, and curcumin + omega-3 group, COX IV and Tomm20 positive areas, both mitochondrial markers, were measured. The curcumin + omega-3 group showed larger positive areas than the curcumin group. This indicates that the combined administration of curcumin and omega-3 is more effective for the recovery of mitochondrial function.

Despite the promising findings of our study, several limitations should be acknowledged. First, our experiments were primarily conducted using an MIA-induced OA rat model and in vitro analyses with human chondrocytes, which may not fully recapitulate the complexity of human osteoarthritis in clinical settings. Second, although we demonstrated the beneficial effects of combined curcumin and omega-3 administration, the precise molecular mechanisms underlying their synergistic action on mitochondrial function remain to be elucidated. Third, the duration of treatment and follow-up in our animal model was relatively short, and the long-term effects and safety profiles require further investigation. Finally, although our results are encouraging, clinical trials are necessary to confirm the efficacy and safety of this combination therapy in human OA patients.

Our study demonstrated that the combined administration of curcumin and omega-3 regulates pain and inhibits cartilage destruction more effectively than a single administration of curcumin or NSAIDs. The results of our study are meaningful, as no previous studies have been conducted on the combined administration of curcumin and omega-3 or its therapeutic effect. Also, no previous study has confirmed the ability of omega-3 to inhibit the catabolic response in human chondrocytes, as confirmed in this study. This study alone cannot clearly explain how the combined administration of curcumin and omega-3 restores mitochondrial dysfunction; however, the combined administration was shown to be more effective than the single administration of curcumin, an important outcome.

## Conclusion

Curcumin and omega-3 are medicines extracted from food, and their stability has been clinically proven. They also have less adverse effects than other medicines. As the combined administration of the two medicines with proven stability has been found to be effective, there would be few restrictions when conducting a clinical trial. Therefore, additional studies should be conducted. We expect that the combination of curcumin and omega-3 will soon be used as a treatment for inhibiting OA progression through improvement of mitochondrial function.

## Data Availability

The data used to support the findings of this study are included within the article. The data and materials in the current study are available from the corresponding author on reasonable request.

## Abbreviations

OA: osteoarthritis
MMP: matrix metalloproteinase
MIA: monosodium iodoacetate
ROS: reactive oxygen species
OARSI: Osteoarthritis Research Society International
PWL: Paw withdrawal threshold
PWT: paw withdrawal latency
IL: interleukin
Nrf2: nuclear factor-erythroid-2-related factor 2
